# Genetic epidemiology of glioma

**DOI:** 10.1054/bjoc.2000.1612

**Published:** 2001-02

**Authors:** B Malmer, L Iselius, E Holmberg, A Collins, R Henriksson, H Grönberg

**Affiliations:** Department of Radiation Science Oncology, Umeå University Hospital; Department of Surgery, Karolinska Hospital, Stockholm, Sweden; Department of Clinical Genetics, Umeå University Hospital, Sweden; Department of Human Genetics, Southampton General Hospital, Southampton, UK

**Keywords:** familial, glioma, astrocytoma, segregation analyses, hereditary, autosomal recessive

## Abstract

The present study performed a segregation analysis of a cohort of first-degree relatives (FDR) of glioma patients. The families with two or more gliomas were also expanded to determine if any more gliomas could be detected, and if any other types of cancers were associated. These glioma-prone families (*n* = 24/432) were extended to include first-, second- and third-degree relatives (*n* = 807) and a cohort was assembled, the standardized incidence risk for other types of cancer calculated and the pedigrees investigated for a possible mode of inheritance. A segregation analysis of the 2141 FDR in 297 families, performed using the Pointer software, did not clearly reject a multifactorial model χ^2^ (3) = 6.13,*P*< 0.2. However, when letting all parameters be free, the recessive model provided the best fit. In the extended families, no increased risk of other types of cancer was found. This population-based study proposes that familial glioma occurs in about 5% of all glioma cases and that 1% have a possible autosomal dominant inheritance. This first segregation analysis performed in familial glioma must be cautiously interpreted, but an autosomal recessive gene provided the best fit, which could possibly explain 2% of all glioma cases. © 2001 Cancer Research Campaign http://www.bjcancer.com


British Journal of Cancer (2001) **84**(3), 429–434
© 2001 Cancer Research Campaign
doi: 10.1054/ bjoc.2000.1612, available online at http://www.idealibrary.com on IDEAL®

http://www.bjcancer.com

### Genetic epidemiology of glioma

**B Malmer[1], L Iselius[2], E Holmberg[3], A Collins[4], R Henriksson[1] and H Grönberg[1]**

[1]Department of Radiation Science, Oncology, Umeå University Hospital, [2]Department of Surgery, Karolinska Hospital, Stockholm, Sweden, [3]Department of Clinical Genetics, Umeå University Hospital, Sweden, [4]Department of Human Genetics, Southampton General Hospital, Southampton, UK

**Summary** The present study performed a segregation analysis of a cohort of first-degree relatives (FDR) of glioma patients. The families with two or more gliomas were also expanded to determine if any more gliomas could be detected, and if any other types of cancers were associated. These glioma-prone families ($n$ = 24/432) were extended to include first-, second- and third-degree relatives ($n$ = 807) and a cohort was assembled, the standardized incidence risk for other types of cancer calculated and the pedigrees investigated for a possible mode of inheritance. A segregation analysis of the 2141 FDR in 297 families, performed using the Pointer software, did not clearly reject a multifactorial model $\chi^2$ (3) = 6.13, $P < 0.2$. However, when letting all parameters be free, the recessive model provided the best fit. In the extended families, no increased risk of other types of cancer was found. This population-based study proposes that familial glioma occurs in about 5% of all glioma cases and that 1% have a possible autosomal dominant inheritance. This first segregation analysis performed in familial glioma must be cautiously interpreted, but an autosomal recessive gene provided the best fit, which could possibly explain 2% of all glioma cases. © 2001 Cancer Research Campaign http://www.bjcancer.com

**Keywords**: familial; glioma; astrocytoma; segregation analyses; hereditary; autosomal recessive

Since one of the first reports of familial glioma, in a mother and two children (Koch, 1949), there have been many case reports of the aggregation of glioma in families. In our view, familial glioma is defined as a first- (FDR) or second-degree relative (SDR) with glioma in the family except the proband. A case-control study showed an increased risk of developing primary brain tumours (PBT) among FDR to glioma patients (Wrensch et al, 1997). In a cohort study, we found a threefold increased risk for FDR to glioma patients to develop glioma (Malmer et al, 1999). In some families there is a known inherited syndrome that explains the cluster of glioma, as in neurofibromatosis (NF) and the Li-Fraumeni syndrome (Louis and von Deimling, 1995), but in many cases of familial glioma no predisposing syndrome is known. The only established environmental risk factor, apart from these genetic syndromes, is ionizing radiation (Karlsson et al, 1998). Beyond this, the aetiology of brain tumours is poorly understood.

Previously, segregation analyses of breast cancer have supported an autosomal dominant gene in families with a hereditary breast cancer (Iselius et al, 1991). These findings have been confirmed and two breast cancer susceptibility genes have been identified, *BRCA 1* and *2*, showing an autosomal dominant inheritance (Wooster et al, 1994). Segregation analyses in colorectal and prostate cancer also support dominant inheritance of these cancers in some families (Houlston et al, 1995; Gronberg et al, 1997). In testicular cancer, a segregation analysis has supported an autosomal recessive gene (Heimdal et al, 1997) and a recent linkage study has mapped a testicular cancer locus to Xq27 (Rapley et al, 2000). However, no segregation analysis has been performed on adult familial glioma.

*Received 17 August 2000*
*Revised 29 October 2000*
*Accepted 1 November 2000*

*Correspondence to:* B Malmer

We used complex segregation analysis on a population-based material of FDR to patients with glioma to study the mode of inheritance in familial glioma (Malmer et al, 1999). An attempt was also made to identify additional cases of glioma in an extended cohort of glioma-prone families and to compare the extended pedigrees, including first; second- and third-degree relatives, with the result of the segregation analysis. The occurrence of other types of cancer associated with these families was also investigated.

### MATERIAL AND METHODS

### Families

Between 1985 and 1993, 463 cases of astrocytoma, regarded in this study as equivalent to glioma were diagnosed in the northern region of Sweden. A questionnaire asking for names of first-degree relatives (FDR) and second-degree relatives (SDR), date of birth and death, and if the relatives had any type of cancer was mailed to 432 of these cases, 31 having been excluded for various reasons (Malmer et al, 1999). 297 (68%) responses to the questionnaire were received. The mean age of the respondents was 53 years. There were no statistically significant differences between respondents and non-respondents in type of astrocytoma, self or proxy-reports, sex or median age (see Malmer et al, 1999).

### Segregation analysis

The 297 respondents had 2141 FDR (Malmer et al, 1999) and the complete personal identification number (ID) was identified in 1890 (88%) but in 203 (9.5%) this was not found, but the name and year of birth and death was known. These cases were included in the analysis and coded as unaffected since they had been stated as unaffected in the questionnaire. In 14 families there were 16 FDR with a history of glioma, which was verified through medical

**Table 1** Each person in the population-based segregation analysis of first-degree relatives (FDR) to patients with glioma was assigned to one of 9 liability classes since the risk of being affected varies with age and sex

| Men/Women (age) | Cumulative incidence (Ij) | Disease specific mortality (Mj) | Morbid risk (Rj) | Liability class (LI) |
|---|---|---|---|---|
| 0–9/0–9 | 0.000031 | 0 | 0.000031 | 1 |
| 10–24/10–29 | 0.000059 | 0.00003 | 0.000085 | 2 |
| 25–34/30–39 | 0.000102 | 0.000012 | 0.000143 | 3 |
| 35–44/40–49 | 0.000158 | 0.000034 | 0.000239 | 4 |
| 45–54/50–64 | 0.000246 | 0.000084 | 0.00042 | 5 |
| 55–64/65–74 | 0.000342 | 0.000186 | 0.00063 | 6 |
| 75+ (women only) | 0.000373 | 0.000298 | 0.00074 | 7 |
| 65–74 (men only) | 0.000443 | 0.000309 | 0.00088 | 8 |
| 75+ (men only) | 0.000562 | 0.000438 | 0.00111 | 9 |

and pathology records. The complex segregation analysis was performed with the POINTER software (Morton et al, 1983). Since Pointer cannot accept pedigrees, the 297 families were divided into 504 nuclear families, one subset of nuclear families including siblings and parents of the proband and one subset of nuclear families including spouse and children of the proband. It was thus a mixture of complete and incomplete selection, since ascertainment through both parents and children was used. Data for the 257 wives or husbands could not be collected from the questionnaire and were coded as unaffected and having the same age as the proband.

The segregation analysis was performed under the mixed model, which assumes the familial aggregation to be due to a major gene, a multifactorial component and a random environmental component, each acting independently. The estimated parameters are: H = multifactorial heritability; q = the estimated gene frequency of the major locus in the population; t = displacement between the homozygous means in standard deviations; d = degree of dominance, where d = 0 corresponds to an autosomal recessive gene and d = 1 corresponds to an autosomal dominant gene. Each person was assigned to one of nine liability classes since the risk of being affected varies with age (Table 1). The risk of being affected was defined by Iselius et al (1991), as indicated by the formula:

$$R_j = (I_j - M_{j-i}/(I - M_{j-i})$$

$R_j$ is the risk of being affected (morbid risk), $I_j$ is the cumulative incidence to the midpoint of the jth age interval and $M_{j-i}$ is the disease-specific mortality to the end of the preceding age interval. The incidence and mortality rates for glioma in each class (Ij) were calculated from the Regional Cancer Registry for Northern Sweden during the years 1958–1994. The age of each case was set by time at ascertainment, age at developing a glioma or age at death. Both joint and conditional likelihood were used when testing the competing hypotheses, calculating the difference of minus twice the log likelihood (-2lnL + C) between the general model and the reduced model, thereby getting a $\chi^2$ distribution. The degrees of freedom were equal to the difference in free parameters between the models. There was one pointer in the material of two brothers both being probands in the study.

### Analyses of 24 extended PBT families

PBTs were found in 24 families of these 297 glioma cases and these had the following characteristics:

1. at least two FDR with glioma (*n* = 14)
2. one second-degree relative except for the proband with glioma (*n* = 8)
3. two relatives with PBT (excluding glioma) other than the proband in the family (*n* = 2).

A number of families with only one third-degree relative except the proband were also identified but they were considered as probably being in the same family due to chance only and were excluded from further analyses. Additional family members in the selected families (*n* = 24) were searched for through the Regional Archives to obtain the names and the unique 10 digit personal ID number of all first-degree relatives, uncles, aunts, cousins, and in some families cousins' children. We identified 1752 relatives in these 24 families and of these 313 (18%) died before 1958 or emigrated and were therefore excluded from further analysis, as linkage to the Cancer Registry was not possible in these cases. A date of death was known for these 313 persons and in most cases also the cause of death. In addition, 41 persons (2.3%) having an unknown date of death and born before 1885 were also excluded since they had probably died before 1958. The remaining 1398 relatives were then linked to the nation-wide Swedish Cancer Registry to identify all cancer diagnoses in the period 1958–1994. If a history of PBT was recorded in the questionnaire or the registry linkage, an independent confirmation was made through medical and histopathology records, thereby confirming the diagnoses made before 1958 when the Swedish Cancer Registry was established.

For the study of other malignancies than PBT among the relatives, a cohort was constructed of all first-degree relatives (FDR) and all uncles, aunts, and cousins in the 24 selected families. The 24 probands and 29 family members with PBT, which had been selected for, were excluded from the cohort analysis. The children of cousins were only known in a small number of the families and were therefore excluded from this analysis (*n* = 562). The remaining 807 relatives were linked to the Swedish Cancer Register, the National Population Registry (SPAR), and to the Swedish Causes of Death Register, thereby obtaining current addresses of living cases and date of death of deceased cases, and thus making calculation of person years possible. The numbers of first, second- and third-degree relatives are presented in Table 2.

The National Cancer Registry and the Swedish Causes of Death Register was established in 1958 and 1952 respectively. Person-years were therefore calculated from 1 Jan 1958 to 31 December 1994 using the software PYRS (Coleman et al, 1989). The cancer incidence rates for the northern region of Sweden for the period 1958–1994 were obtained from the Regional Cancer Registry since

 *© 2001 Cancer Research Campaign*

**Table 2** Number of relatives included and excluded from the 24 families in the cohort analysis with at least 2 glioma or three or more primary brain tumours (PBT) in the family

| Type of relative | Included in analysis | Excluded because of death before 1958 or emigrated | Excluded because of unknown date of death | Included and excluded persons |
|---|---|---|---|---|
| First-degree relatives | 174 (90.2%) | 18 (9.3%) | 1 (0.5%) | 193 (100%) |
| Second-degree relatives (uncles and aunts) | 138 (52.7%) | 109 (41.6%) | 15 (5.7%) | 262 (100%) |
| Third-degree relatives (cousins) | 495 (77.6%) | 128 (20.1%) | 15 (2.3%) | 638 (100%) |
| All relatives | 807 (73.8%) | 255 (23.3%) | 31 (2.9%) | 1093 |

the relatives in the cohort were mainly residents of this region. The expected number of cases was calculated by multiplying these incidence rates by calendar and age-specific person-years. The standardized incidence ratio (SIR) was defined as the ratio between the observed and expected number of cases. Exact confidence limits of the SIR were calculated using the formula suggested by Byar (Breslow and Day, 1980). Survival among the astrocytoma patients was calculated from the date of diagnosis to the date of death or follow-up to 1 October 1998 if the patients were alive.

### RESULTS

In our population-based cohort study, 22 of 432 initially included families (5%) were identified with an aggregation of glioma. The segregation analysis includes 297 families of which 14 families had at least two FDR affected with glioma. Among those 14 families, 3 (21%) have a suspected dominant mode of inheritance in the expanded pedigree, 9 (65%) have affected siblings only, favouring an autosomal recessive gene, and 2 (14%) have parent–child pairs.

### Segregation analysis

The result of the segregation analysis is presented in Table 3. When calculated under joint likelihood, the familial aggregation of glioma was not due to chance, since the sporadic model was strongly rejected $\chi^2$ (4) = 110.18, $P < 0.001$. The multifactorial model was not clearly significantly rejected in favour of a major gene $\chi^2$ (3) = 6.13, $P < 0.2$. However, when testing for the best model letting all parameters be free, the recessive model provided the best fit for the generalized major locus, and the dominance at the locus approached zero (Table 3). When calculated under conditional likelihood, the sporadic model was strongly rejected, $\chi^2$ (3) 83.29, $P < 0.001$, but the segregation analysis did not provide strong evidence for a single locus.

### Extended pedigree analyses

The 3 families with three or more glioma patients in the pedigree are presented in Figure 1. In the most extreme case, there were 5 gliomas and one ependymoma with 4 gliomas on the same side of the family (family 1). In family 2, three siblings had glioma, all developing the disease at an early age (13, 25, 40 years). There were also two children with soft tissue sarcoma in the family, thereby fulfilling the criteria for Li Fraumeni syndrome (Birch et al, 1994). A mother affected with both glioma and amyotrophic lateral sclerosis (ALS) had two children who developed glioma in their mid-thirties (family 3). The number of relatives affected with PBT in families 1–3 and the characteristics and diagnoses of the family members are listed in Table 4. Among the families with only two members with glioma, there were two parent–child pairs, 9 pairs of siblings, and 8 families with the proband and a second-degree relative. The overall risk of developing other cancers than PBT in the 24 families was not increased, SIR 0.88 (95% CI 0.72–1.07) with 101 observed and 114.7 expected cases. In addition, no individual cancer site showed an increased or decreased significant risk.

### DISCUSSION

The findings of this, the first segregation analysis of familial glioma, favours an autosomal recessive gene. This model provided the best fit, although the multifactorial model was not clearly rejected and the results must therefore be interpreted cautiously. There is a well documented increased risk of FDR of glioma patients developing glioma (Wrensch et al, 1997; Malmer et al, 1999). In our study, the glioma families are heterogeneous, with a

**Table 3** Segregation analysis of first-degree relatives to probands with astrocytoma grade I–IV diagnosed 1985–1993 in northern Sweden, calculated under joint likelihood

| Model | Multifactorial heritability | Gene frequency in the population | Displacement between means for homozygous individuals | Dominance at the major locus | −2lnL+C |
|---|---|---|---|---|---|
| Sporadic | (0) | (0) | – | – | 481.99 |
| Multifactorial | 0.68 | (0) | – | – | 377.94 |
| Dominant | (0) | 0.000388 | 2.10 | (1) | 381.07 |
| Additive | (0) | 0.020 | 3.11 | (0.5) | 375.42 |
| Recessive | (0) | 0.026 | 2.58 | (0) | 374.91 |
| Generalized single locus | (0) | 0.014 | 3.05 | 0.33 | 373.07 |
| General model (heritability parameter free) | 0.517 | 0.022 | 2.31 | 0 | 371.81 |

() = degrees of freedom.

© 2001 Cancer Research Campaign mixture of sibships, pedigrees with an autosomal dominant picture and some families with two astrocytomas in two generations. However, the majority (65%) of the families with FDR are siblings. Inherited glioma-prone syndromes that are autosomal dominantly inherited are the Li-Fraumeni syndrome and NF1 and NF2, but siblings are often described in the literature. In a summary of case reports, 18 cases of siblings or twins with primary brain tumours (PBT) were reported and in five cases with

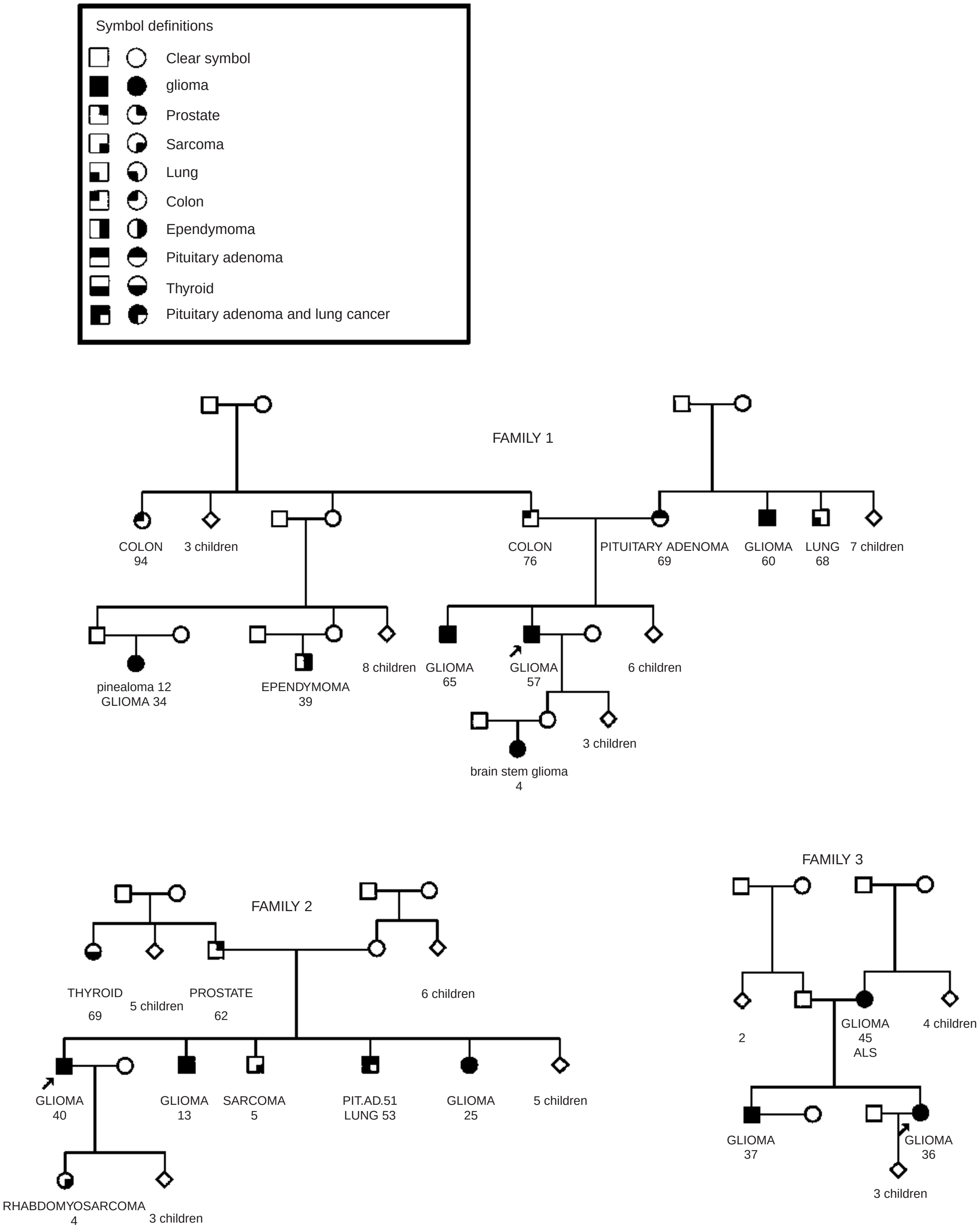

**Figure 1** Three families with a suspected dominant mode of inheritance among the 24 glioma-prone families. ALS = Amyotrophic lateral sclerosis

 *© 2001 Cancer Research Campaign*

**Table 4** Characteristics of primary brain tumours in family members in family 1–3, with three or more astrocytoma among first- and second-degree relatives. Age at diagnosis, survival and the basis of diagnosis

| Family number | Pedigree number | Diagnosis | Basis of diagnosis | Age at diagnosis (y) | Survival (months) | Sex |
|---|---|---|---|---|---|---|
| 1 | III:1 | astro grIII | HP | 40 | 42 | M |
| | III:2 | glioma | HP | 13 | 9 | M |
| | III:3 | glioma | X-ray | 25 | 9 | F |
| 2 | II:6 | astrogr III–IV | HP | 60 | 8 | M |
| | III:4 | astro III–IV | HP | 65 | 6 | M |
| | III:5 | astro III–IV | HP | 57 | 6 | M |
| | V:1 | brain stem glioma | CT | 4 | 8 | F |
| | IV:1 | pinealoma | X-ray | 12 | | |
| | | astrocytoma I | HP | 34+ | 160 | F |
| | IV:2 | ependymoma | HP | 36 | 16 | M |
| 3 | II:3 | astro gr I | HP | 45 | 56 | F |
| | III:1 | astro gr II–III | HP | 37+ | 15 | M |
| | III:2 | astro gr II–III | HP | 36+ | 66 | F |

HP = histopathologically verified; CT = diagnosed with computed thomography; X-ray = diagnosed with ventriculography; + = patient alive; astro = astrocytoma.

PBT in two generations (Vieregge et al, 1987). A recent study also found a majority of siblings but regarded environmental agents as possible causes (Grossman et al, 1999) so other as yet unidentified genetic and environmental factors must be also considered in glioma aetiology. An autosomal recessive gene could be part of the explanation of the sibpair excess.

When segregation analysis favours an autosomal recessive gene, certain biases must be considered. First, there could be ascertainment bias if there is a lack of information of affected status in the parental generation. In our families, all parents in the glioma-prone families were ascertained through the Swedish Cancer Registry. Second, one must consider affected status may influence fertility, as in a testicular cancer study (Heimdal et al, 1997). This is not an issue in our study since the median age at disease development of the probands was 57 years for glioma. Third, an environmental agent may be responsible for the aggregation of glioma cases. However, there is no established aetiologic factor for glioma except ionizing radiation (Karlsson et al, 1998), which explains only a few cases of glioma. There are no such obvious sources of bias in this study. The advantage of segregation analysis is that the gene frequency of the population can be calculated in population-based material. It can also adjust for a multifactorial component; in this study the findings may favour a major autosomal recessive gene.

This segregation analysis was performed under both conditional and joint likelihoods. Conditional likelihood strongly rejected the hypothesis that the aggregation could be due to chance, although no obvious gene model was favoured. Nevertheless, when calculating under joint likelihood, an autosomal recessive gene provided the best fit and was on the border of significance. A study testing different hypotheses showed a substantial drop in power when testing the hypotheses for a recessive gene under conditional likelihood compared to joint likelihood (Borecki et al, 1994). Since our material is rather small, this could explain the differences between conditional and joint likelihoods. A previous segregation analysis of childhood brain tumours, calculating the segregation for cancer over-all in these families, found that the multifactorial model provided the best fit (Bondy et al, 1991). Given our rather small number of families, this segregation analysis needs to be confirmed in a larger cohort.

In two families, the extended pedigree identified more cases of glioma, indicating a different mode of inheritance compared to the interpretation in the segregation analysis, which comprised only first-degree relatives. The criteria for Li-Fraumeni syndrome is fulfilled in family 2, since, apart from three siblings with glioma, there was a brother with sarcoma and also a child to the proband with rhabdomyosarcoma (Figure 1). In addition, family 1 was coded as only two siblings in the segregation analysis, whereas in the expanded pedigree it had a suspected maternal dominant mode of inheritance with reduced penetrance. Apart from these two families, no differences were found between the segregation analysis and the expanded pedigrees.

In addition, the medical records of the affected in the glioma-prone families were reviewed for signs of NF1 or other inherited syndromes that could explain the familial aggregation. No positive family history of associated diseases or inherited disorders was found. Interestingly, among other cancer diagnoses in the extended family pedigrees, pituitary adenoma was apparent in both a brother to the proband in family 1 and a mother of the proband of family 2. To our knowledge, this has not been described previously in glioma families. There was no site-specific significant increased risk for any type of cancer in the cohort analysis. Clearly, our families do not co-segregate with other inherited cancers sites, such as breast or colon cancer.

Similarities within families can be seen in Table 4. On the maternal side of family 2, all family members had an aggressive tumour and short survival, whereas in family 3 all members had a low-grade glioma and a long survival. This should be considered in light of the fact that astrocytoma patients in general have a median survival of about 12 months. An interesting observation is that families 1 to 3 aggregate in the most northern county of Sweden, Norrbotten. Our genealogical research has currently not shown any relation between the families, but if the families are remotely related to each other, a founder mutation is a possibility.

In conclusion, this population-based study suggests that aggregation of glioma occurs in about 5% (22/432) of all families to glioma patients. This can be compared with 10–15% in familial breast cancer (Rosenthal and Puck, 1999) and 20% in colorectal cancer (Prichard and Tjandra, 1998). It is likely that familial glioma is a heterogenic disease with different causes. Our study suggests a dominant mode of inheritance in about 1% of all glioma cases, and if there is a dominant trait, that it is not fully penetrant. In the segregation analysis, an autosomal recessive gene model provides the best fit, which could possibly explain 2% of all

*© 2001 Cancer Research Campaign* 

gliomas. In the glioma-prone families analysed, no increased risk of other malignancies was detected.

### ACKNOWLEDGEMENTS

This study was supported by grants from the Lion's Cancer Foundation and the unit of Oncology at the Department of Radiation Science, Umeå University. Mrs Karin Andersson is acknowledged for excellent secretarial assistance.

### REFERENCES

Birch JM, Hartley AL, Tricker KJ, Prosser J, Condie A, Kelsey AM, Harris M, Jones PH, Binchy A and Crowther D (1994) Prevalence and diversity of constitutional mutations in the p53 gene among 21 Li-Fraumeni families. *Cancer Res* **54**: 1298–1304

Bondy ML, Lustbader ED, Buffler PA, Schull WJ, Hardy RJ and Strong LC (1991) Genetic epidemiology of childhood brain tumours. *Genetic Epidemiol* **8**: 253–267

Borecki IB, Province MA and Rao DC (1994) Power of segregation analysis for detection of major gene effects on quantitative traits. *Genetic Epidemiol* **11**: 409–418

Breslow N and Day N (1980) *Statistical methods in cancer research. The analysis of case control studies*. IARC, Lyon 1

Coleman MP, Hermon C and Douglas A (1989) Person Years (PYRS), a fortran program for cohort study analysis; IARC report 89/006 International Agency for Research of Cancer. *IARC Publications*

Gronberg H, Damber L, Damber JE and Iselius L (1997) Segregation analysis of prostate cancer in Sweden: support for dominant inheritance. *American Journal of Epidemiol* **146**: 552–557

Grossman SA, Osman M, Hruban R and Piantadosi S (1999) Central nervous system cancers in first-degree relatives and spouses. *Cancer Investigation* **17**: 299–308

Heimdal K, Olsson H, Tretli S, Fossa SD, Borresen AL and Bishop DT (1997) A segregation analysis of testicular cancer based on Norwegian and Swedish families. *British Journal of Cancer* **75**: 1084–1087

Houlston RS, Collins A, Kee F, Collins BJ, Shields DC and Morton NE (1995) Segregation analysis of colorectal cancer in Northern Ireland. *Human Heredity* **45**: 41–48

Iselius L, Lambert B, Lindsten J, Tippett P, Gavin J, Daniels G, Yates A, Ritzen M and Sandstedt B (1979) Unusual xx/xy chimerism. *Annals of Human Genetics* **43**: 89–96

Iselius L, Slack J, Littler M and Morton NE (1991) Genetic epidemiology of breast cancer in Britain. *Annals of Human Genetics* **55**: 151–159

Karlsson P, Holmberg E, Lundell M, Mattsson A, Holm LE and Wallgren A (1998) Intracranial tumours after exposure to ionising radiation during infancy: a pooled analysis of two Swedish cohorts of 28,008 infants with skin hemangioma. *Radiation Res* **150**: 357–364

Koch G (1949) Erbliche Hirngeschwulste. *Zeitschrift für Menschliche Vererbungs- und Konstitutionslehre* **29**: 400–423

Louis DN and von Deimling A (1995) Hereditary tumour syndromes of the nervous system: overview and rare syndromes. *Brain Pathology* **5**: 145–151

Malmer B, Gronberg H, Bergenheim AT, Lenner P and Henriksson R (1999) Familial aggregation of astrocytoma in northern Sweden: an epidemiological cohort study. *International Journal of Cancer* **81**: 366–370

Morton NE, Rao DC and Lalouel JM (1983) Methods in genetic epidemiology. In *Contributions to epidemiology and biostatistics*, ed, Karger, pp. 62–98. Basel: Prichard PJ and Tjandra JJ (1998) Colorectal cancer. *Medical Journal of Australia* **169**: 493–498

Rapley EA, Crockford GP, Teare D, Biggs P, Seal S, Barfoot R, Edwards S, Hamoudi R, Heimdal K, Fosså S, Tucker K, Donald J, Collins F, Friedlander M, Hogg D, Goss P, Heidenreich A, Ormiston W, Daly PA, Forman D, Oliver RTD, Leahy M, Huddart R, Cooper CS, Bodmer JG, Easton DF, Stratton MR and Bishop DT (2000) Localization to Xq27 of a susceptibility gene for testicular germ-cell tumours. *Nature Genetics* **24**: 197–200

Rosenthal TC and Puck SM (1999) Screening for genetic risk of breast cancer. *American Family Physician* **59**: 99–104

Vieregge P, Gerhard L and Nahser HC (1987) Familial glioma: occurrence within the "familial cancer syndrome" and systemic malformations. *Journal of Neurology* **234**: 220–232

Wooster R, Neuhausen SL, Mangion J, Quirk Y, Ford D, Collins N, Nguyen K, Seal S, Tran T and Averill D (1994) Localization of a breast cancer susceptibility gene, *BRCA 2*, to chromosome 13q12–13. *Science* **265**: 2088–2090

Wrensch M, Lee M, Miike R, Newman B, Barger G, Davis R, Wiencke J and Neuhaus J (1997) Familial and personal medical history of cancer and nervous system conditions among adults with glioma and controls. *American Journal of Epidemiol* **145**: 581–593

 © 2001 Cancer Research Campaign
